# Dispersion of Pt Nanoparticle-Doped Reduced Graphene Oxide Using Aniline as a Stabilizer

**DOI:** 10.3390/ma5122927

**Published:** 2012-12-18

**Authors:** Ali Grinou, Young Soo Yun, Se Youn Cho, Hyun Ho Park, Hyoung-Joon Jin

**Affiliations:** Department of Polymer Science and Engineering, Inha University, Incheon 402-751, Korea; E-Mails: arib_44@hotmail.com (A.G.); ysyun@inha.edu (Y.S.Y.); 01n015@inha.edu (S.Y.C.); hhpark@inha.edu (H.H.P.)

**Keywords:** graphene oxide, aniline, Pt nanoparticles, hybrid, stabilizer

## Abstract

In this study, a simple one-step method was developed to load small-sized Pt nanoparticles (3.1 ± 0.3 nm) in large quantities (50 wt %) on aniline-functionalized and reduced graphene oxide (r-fGO). In the process, an ethylene glycol solution and aniline-functionalized moiety play the roles of reducing agent and stabilizer for the Pt nanoparticles, respectively, without damaging the graphite structures of the r-fGO. The Pt nanoparticles loading on the surface of r-fGO with uniform dispersion have a great effect on the electrical conductivity.

## 1. Introduction

Metal nanoparticles have attracted considerable interest, because of their unique performance in electronic, magnetic, optical and catalytic applications and many other fields [1]. The specific activity of catalysts is strongly related to their size, distribution and support. Highly distributed catalyst particles with a small size and narrow size distribution are ideal for high electrocatalyst activity, due to their large surface-to-volume ratio. Among the possible supports, carbon black and carbon nanotubes with dispersed metal nanoparticles (NPs) have been widely used as electrodes [2]. Recently, graphene, a new two-dimensional nanomaterial composed of sp^2^-bonded carbon atoms, has attracted a great deal of attention due to its unique nanostructure and excellent properties, such as high mechanical strength (>1060 GPa), high thermal conductivity (~3000 W/mK), high electron mobility (15,000 cm^2^/Vs) and high specific surface area (2600 m^2^/g) [3,4]. These remarkable characteristics make it a promising candidate as a new 2D support for metal NPs, such as Pt, Au, Pd, *etc.* [5]. It is expected that the metal NPs anchored on graphene sheets could potentially exhibit novel catalytic, magnetic and optoelectronic properties. In particular, platinum nanoparticles (Pt NPs) have been subject to intensive research for the design of electrodes [6], and Pt is an important catalyst for many chemical and electrochemical reactions, including oxygen reduction, hydrogen oxidation, methanol oxidation and hydrogenations [7]. Well-dispersed, small-sized Pt NPs are expected to exhibit enhanced activity and selectivity for catalytic reactions [8]. Recently, a platinum hybrid supported by graphene oxide (GO) has attracted attention for its promising potential application in catalysis for fuel cell reactions, sensors and gas storage [9,10,11,12,13,14]. GO has been used successfully as a host layered material to prepare hybrids of reduced GO and metal NPs, due to its easy exfoliation and excellent intercalation properties. During the past decades, researchers have made considerable efforts and developed preparations of size-controlled spherical metal NPs along with their assemblies [15]. However, the effective attachment of small-sized Pt NPs dispersed uniformly in large quantities onto the surface of graphene nanosheets remains a great challenge [16]. This paper reports a simple process for preparing well-dispersed Pt NPs with small particle size in large quantities loaded on reduced functionalized graphene oxide (Pt NPs/r-fGO), using aniline as stabilizer. Ethylene glycol (EG) was employed as a reducing agent for the functionalized graphene oxide and Pt NPs, in a single step using a procedure described previously [17]. The main aim was to develop a simple and effective synthetic route that provides well-dispersed Pt NPs with small particle size in large quantities on the surface of reduced graphene oxide (r-GO). Aniline was used as a stabilizer to obtain a uniform dispersion of Pt NPs doped in large quantities onto the surface of r-GO and to control the Pt NP size by preventing agglomeration on the surface of r-GO. Consequently, the use of aniline as a stabilizer for Pt NPs in large quantities enhances the catalytic performance of the hybrid Pt NPs/r-fGO. A morphological investigation by transmission electron microscopy (TEM) showed that small Pt NPs in large quantities were loaded uniformly on the surface of r-GO using aniline as a stabilizer, in contrast to Pt NPs deposited without aniline. This confirms the effect of aniline as a stabilizer for Pt NPs. The function of aniline as a stabilizer plays an important role in loading Pt NPs onto the r-GO surface.

## 2. Results and Discussion

**Figure 1:**
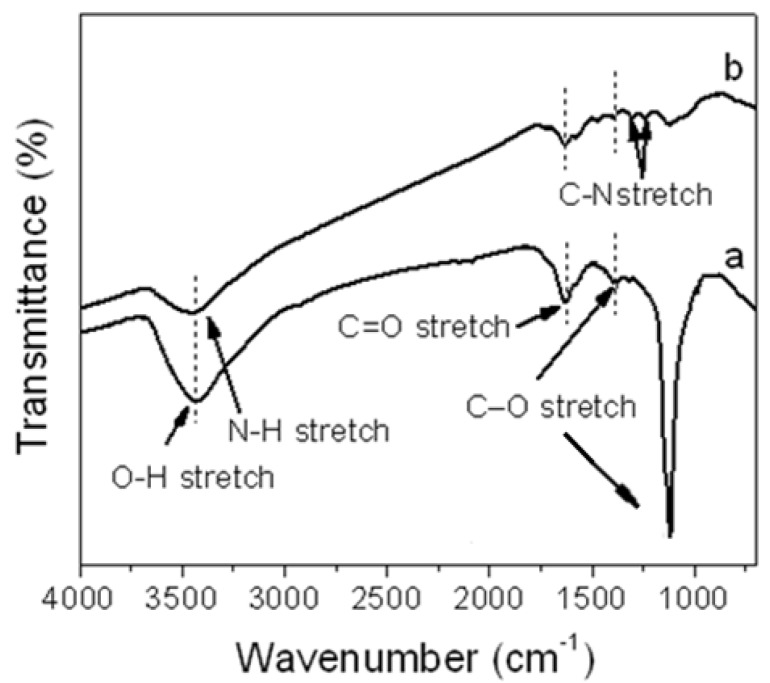
Fourier transform infrared (FT-IR) spectroscopy of (**a**) graphene oxide (GO) and (**b**) functionalized graphene oxide (f-GO).

Figure 1 shows the FT-IR spectra of GO and aniline functionalized graphene oxide (f-GO). In the GO spectrum (Figure 1a), the peaks at 3458, 1624, 1387 and 1116 cm^−1^ were assigned to the O–H stretching, C=O stretching, C–O (carbonyl) and C–O (epoxy/ether) vibrations, respectively. When GO was chemically functionalized by aniline (f-GO), the new peak at 1315 cm^−1^ was assigned to C–N stretching vibrations, confirming the presence of aniline on the GO surface. This confirms the successful functionalization of GO by aniline. Also, the intensity of the peaks for the O–H stretching, C=O stretching and C–O (carbonyl) significantly decreased, and the peak of the C–O (epoxy/ether) vibrations almost disappeared, because of the reduction effect for aniline monomer [18].

**Figure 2:**
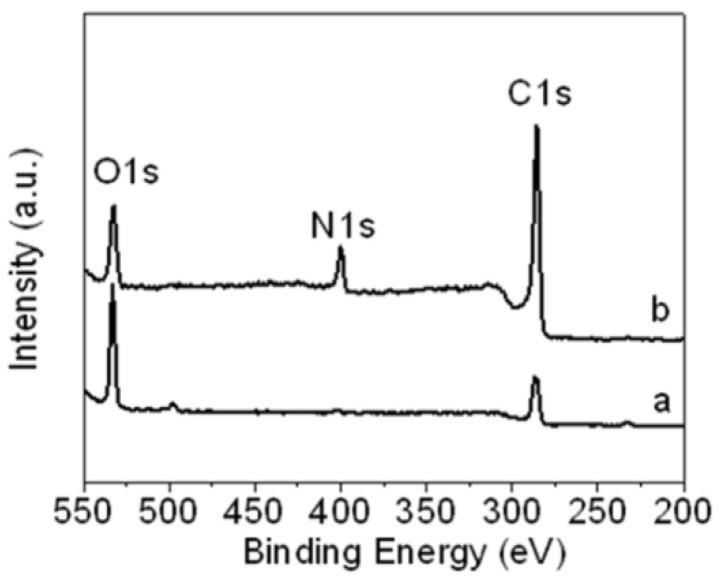
X-ray photoelectron spectroscopy (XPS) of (**a**) GO and (**b**) f-GO.

For more confirmation, X-ray photoelectron spectroscopy (XPS) was used to characterize the N 1s peak of aniline-functionalized GO. Figure 2 shows the XPS of (a) GO and (b) f-GO. The GO only has C 1s and O 1s peaks, which were attributed to the carboxyl and carbonyl groups after the oxidation treatment, as shown in the Figure 2a. After being functionalized by the aniline stabilizer, a new peak appeared at 399.9 eV, which was assigned to the nitrogen band (N 1s), as shown in Figure 2b. The intensity of the carbon (C 1s) peak increased due to the incorporation of aniline carbon, and the intensity of the oxygen group decreased after the GO was functionalized, due to the elimination of oxygen groups during the chemical reaction between GO and aniline, which confirmed the successful functionalization of GO by the aniline stabilizer. This data confirms the results obtained by Fourier transform infrared (FT-IR) spectroscopy.

**Figure 3:**
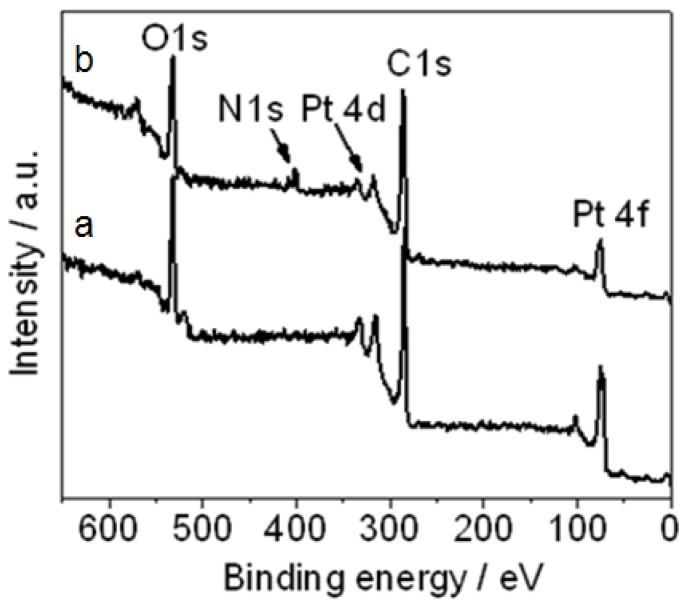
XPS of (**a**) platinum nanoparticles (Pt NPs)/r-GO and (**b**) Pt NPs/r-fGO hybrid.

Figure 3 shows the XPS of (a) Pt NPs/r-GO and (b) Pt NPs/r-fGO in which there were peaks for Pt 4f, C 1s, Pt 4d, N 1s and O 1s in Pt NPs/r-fGO. The N 1s peak at 402.2 eV was observed in the Pt NPs/r-fGO only in the presence of the aniline stabilizer, and this obvious difference was attributed to the nitrogen in the aniline stabilizer. For the aniline-stabilized Pt NPs/r-fGO hybrid, the interaction between aniline and Pt was characterized by the N 1s line of the XPS, as shown in Figure 3a. The peak for N 1s at 402.2 eV demonstrated the presence of nitride bonding (Pt–N) between the head-on N atoms of aniline and the surface Pt atoms of Pt NPs [19]. The benzyl rings surrounding the complexed metal core simultaneously separate the particles from each other and promote the adsorption of Pt NPs onto the surface of the r-GO supports through the π–π interaction. These results confirm the effect of aniline on the stabilization and dispersion of metal NPs on the surface of nanocarbon materials.

**Figure 4:**
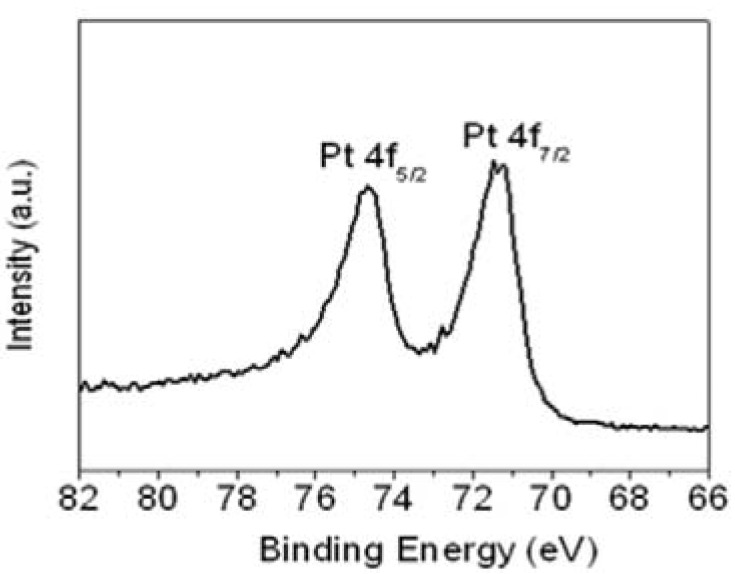
XPS of Pt 4f region for Pt NPs/r-fGO hybrid.

The Pt 4f line in the XP spectrum showed two pairs of peaks from the spin-orbital splitting of the 4f_7/2_ and 4f_5/2_ (Figure 4). The most intense doublets observed at 71.5 and 74.7 eV were assigned to zero-valent Pt (Pt(0)). This demonstrates that well-dispersed Pt(0)-dominated catalysts had been prepared successfully using the aniline stabilizer, and that they were highly dispersed on the surface of r-GO. The morphological structure, particle size and metal dispersion of the Pt NPs on the surface of r-GO were examined by transmission electron microscopy (TEM).

**Figure 5:**
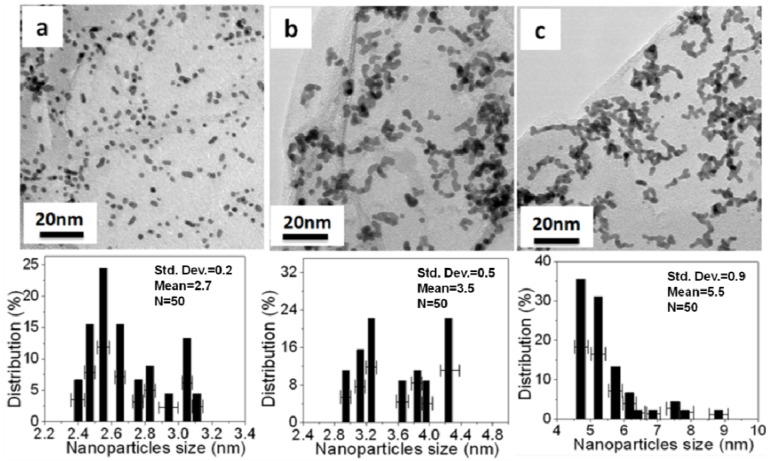
TEM images and histogram of the Pt NPs of Pt NPs/r-GO hybrids in the absence of aniline with different weights of Pt NPs loaded on the r-GO surface: (**a**) 10 wt %; (**b**) 30 wt % and (**c**) 50 wt %.

**Figure 6:**
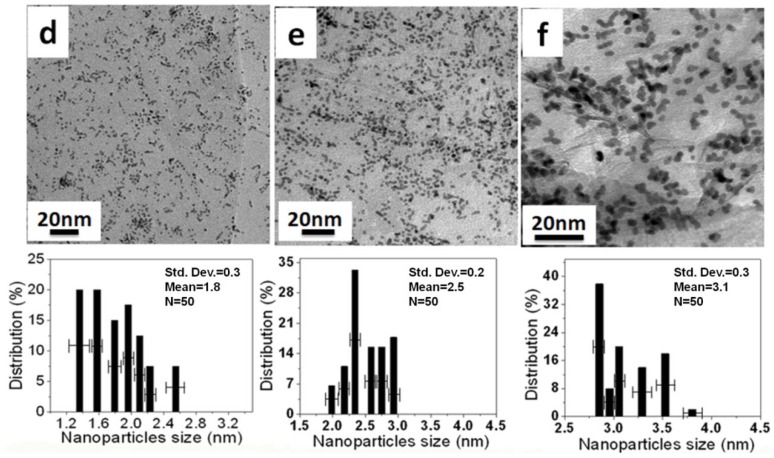
TEM images and histogram of Pt N of Pt NPs/r-fGO hybrids in the presence of aniline as a stabilizer with different weights of Pt NPs loaded on the surface of r-GO: (**d**) 10 wt %; (**e**) 30 wt %; and (**f**) 50 wt %.

Figure 5 and Figure 6 show TEM images and histograms of the metal particle size of Pt NPs/r-fGO and Pt NPs/r-GO, respectively. Fifty particles were measured randomly to obtain the particle size distribution. The Pt NPs loaded on the r-GO surface in the absence of aniline are shown in Figure 5a–c. The Pt NPs on the r-GO are large and not dispersed uniformly, with mean particle sizes of 2.7 ± 0.2, 3.5 ± 0.5 and 5.5 ± 0.9 nm corresponding to the 10 wt %, 30 wt % and 50 wt % Pt NPs, respectively. This was due to the aggregation of the Pt NPs upon heat treatment and the weak interaction between the r-GO and platinum atoms. The Pt NPs, which were prepared using the aniline stabilizer with mean particle sizes of 1.8 ± 0.3, 2.5 ± 0.2 and 3.1 ± 0.3 nm corresponding to 10 wt %, 30 wt % and 50 wt % Pt NPs, respectively, were dispersed uniformly on the surface of r-GO, as shown in Figure 6d–f. These small Pt NPs suggest a strong interaction between r-GO and platinum atoms, because there was no aggregation of the Pt NPs upon heat treatment [20]. Therefore, the function of aniline as a stabilizer plays an important role in loading and controlling the size of the Pt NPs on the surface of r-GO. 

**Figure 7:**
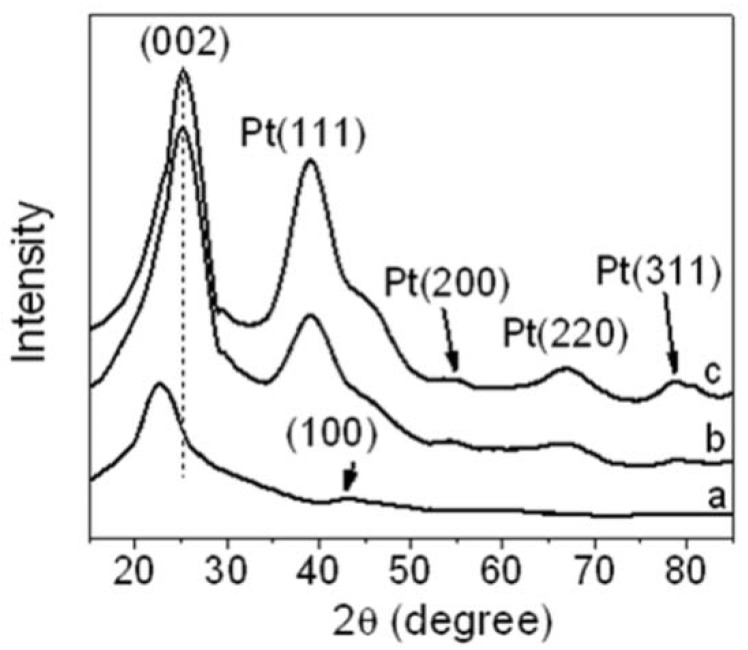
X-ray powder diffraction (XRD) patterns of (**a**) r-GO; (**b**) Pt NPs/r-GO and (**c**) Pt NPs/r-fGO hybrid composite.

Figure 7 shows XRD patterns of r-GO, Pt NPs/r-GO, and Pt NPs/r-fGO. The peak at 23.7° (Figure 7a) was assigned to the characteristic diffraction peak (002) of reduced GO by EG, corresponding to the removal of a large number of oxygen-containing groups. This was attributed to the GO nanosheets being partially reduced to graphene and restacked into an ordered crystalline structure [17]. The diffraction peak at approximately 43° is associated with the (100) plane of the hexagonal structure of carbon [21]. For the Pt NPs/r-GO and Pt NPs/r-fGO hybrid (Figure 7b,c), the position of the (002) diffraction peak (at 23.7°) moved slightly to a higher angle (at 25.4°) after the deposition of Pt NPs on r-GO, which indicates that GO is further converted to the crystalline graphene, and the conjugated graphene network (sp^2^ carbon) has been reestablished by the reduction process. Pt NPs are suggested to play an important role in the reduction of GO when using ethylene glycol as a reducing agent [22]. In addition, crystalline platinum showed strong peaks at 39.2°, 55.0°, 67.3° and 79.8°, which were assigned to the (111), (200), (220) and (311) crystalline planes of face-centered-cubic (fcc) Pt NPs, respectively, indicating a fcc structure. These results confirm that Pt NPs had been dispersed successfully over the surface of functionalized r-GO.

**Figure 8:**
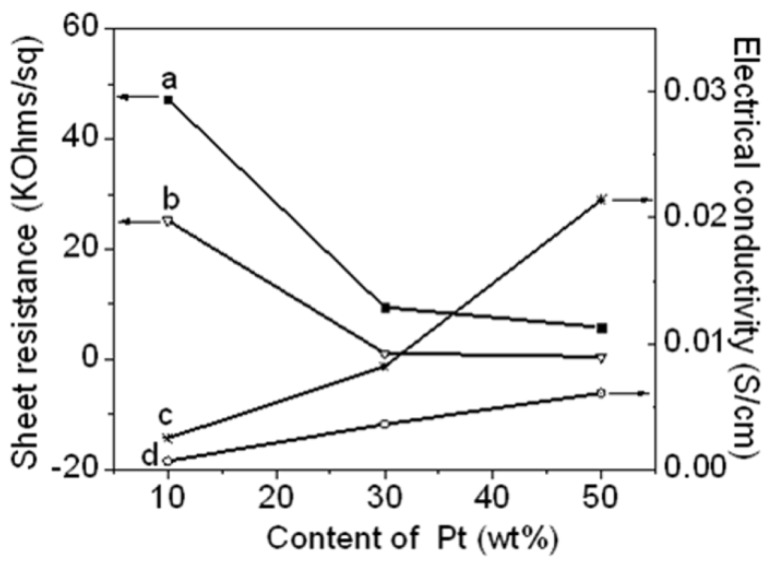
Sheet resistance of (**a**) Pt NPs/r-GO; (**b**) Pt NPs/r-fGO; and electrical conductivity of (**c**) Pt NPs/r-fGO; (**d**) Pt NPs/r-GO as a function of the platinum content in the r-GO hybrids.

**Figure 9:**
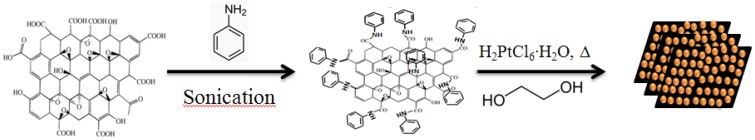
Synthesis of functionalized r-GO/Pt NPs.

Figure 8 shows the sheet resistance and electrical conductivity of the Pt NPs/r-GO and Pt NPs/r-fGO hybrid as functions of the Pt NP content. Figure 9a,d shows the sheet resistance and electrical conductivity of the Pt NPs/r-GO hybrid, respectively. A clear decrease in sheet resistance with increasing platinum content was observed, suggesting that the Pt NP yield improved electrical contact between the r-GO sheets [23]. The sheet resistance decreased from 47.3 to 5.9 KΩ/sq with increasing Pt NP content in the r-GO from 10 to 50 wt %, respectively. Consequently, the electrical conductivities increase from 6.8 × 10^−4^ to 0.6 × 10^−2^ S/cm. The improvement in electrical conductivity might be due to the high Pt NP loading on the surface of the r-GO hybrid. Figure 9b,c shows the sheet resistance and electrical conductivity of the Pt NPs/r-fGO hybrid prepared using the aniline stabilizer, respectively. The sheet resistance in the r-GO decreased from 25.2 to 0.51 KΩ/sq, with Pt NP content increasing from 10 to 50 wt %. Consequently, the electrical conductivity increased from 2.5 × 10^−3^ to 4.6 × 10^−2^ S/cm. The electrical conductivity of the 50 wt % Pt NPs in the r-fGO hybrid prepared using the aniline stabilizer was seven-times higher than that of the 50 wt % Pt NPs in r-GO hybrid prepared without the aniline stabilizer. The difference in electrical conductivity can be related to the morphological behavior, as shown by the TEM images in Figure 6 and Figure 7. The electrical conductivity of the Pt NPs/r-GO hybrid was lower than that of the Pt NPs/r-fGO hybrid, because the electrical contact point is rather insufficient due to the agglomeration of Pt NPs dispersed randomly over the r-GO surface. From this result, the electrical conductivity of the hybrid is strongly dependent on the morphology characteristics, such as the small particle size, loading and highly dispersed Pt NPs on the surface of r-GO.

## 3. Experimental Section

### 3.1. Materials

GO was synthesized from graphite flake purchased from Sigma-Aldrich (Product Number 332461). The aniline monomer was obtained from DC Chemical Co. Ltd. (Korea). Hexachloroplatinic acid (H_2_PtCl_6_), EG, Sulfuric acid (H_2_SO_4_), sodium nitrate (NaNO_3_), potassium permanganate (KMnO_4_) and all other organic solvents used in this study were used without further purification.

### 3.2. Functionalized Graphene Oxide by Aniline Monomer (f-GO)

GO was synthesized using the Hummers method [24]. To prepare the aniline-functionalized graphene oxide, 0.2 g of GO was mixed with 4 g of aniline in 40 mL of Di-water and dispersed by sonication for 4 h. The resulting solution was then washed, filtered and dried at 60 °C for 24 h.

### 3.3. Synthesis of Pt NPs/Functionalized Reduced Graphene Oxide Hybrid (Pt NPs/r-fGO)

The experimental method was exhibited in Figure 9. Pt NPs were loaded on functionalized graphene oxide (f-GO) sheets by the chemical reduction of hexachloroplatinic acid (H_2_PtCl_6_) in an ethylene glycol-water solution. During the reaction, ethylene glycol reduces the f-GO and the chloroplatinic acid in a single step. Using a typical procedure, 50 mg of f-GO in 50 ml of Di-water were sonicated for 15 min to obtain a homogeneous solution. An amount of aqueous H_2_PtCl_6_ solution was added to the above solution and sonicated for another 15 min. To control the incorporated Pt contents on f-GO, 10%, 30% and 50% Pt-doped f-GO were prepared by controlling contents of 2.5 mL, 5.0 mL and 7.0 mL of an aqueous solution of 0.05 M H_2_PtCl_6_·H_2_O, respectively [14]. The mixtures were added to 40 ml of EG in a 250-mL flask. The mixtures were first ultrasonically treated for 4 h to ensure uniform dispersion of H_2_PtCl_6_ and f-GO in the EG-water solution. The reduction reaction was then performed at 100 °C for 24 h with constant stirring. The Pt NPs/r-fGO hybrids were finally separated by filtration and washed several times with DI water. The resulting product was dried under vacuum at 60 °C for 24 h. For comparison, Pt NPs loaded on r-GO without aniline were also produced using the same procedure.

### 3.4. Characterization

Morphological characterization was performed by transmission electron microscopy (TEM, CM200, Philips, Netherlands) to determine the effect of aniline as a stabilizer for the Pt NPs on the surface of r-GO. The TEM samples were prepared by dispersing a small amount of dry powder in ethanol. FT-IR, XPS and FT-Raman spectroscopy were used to characterize the chemical structures of GO before and after functionalization by aniline. X-ray powder diffraction (XRD) was used to determine the crystallinity of r-GO, Pt NPs/r-GO and Pt NPs/r-fGO hybrid. The electrical conductivity and sheet resistance of the GO, f-GO, Pt NPs/r-GO and Pt NPs/r-fGO hybrid were measured using a four-point probe with an electrical conductivity meter (Hiresta-UP MCP-HT450, Mitsubishi Chemical, Japan).

## 4. Conclusions

A simple one-step method was developed to load small-sized Pt NPs (3.1 ± 0.3 nm) in large quantities (50 wt %) on reduced functionalized GO using an EG solution as the reducing agent, and aniline as a stabilizer for the Pt NPs, without damaging the graphite structures of the r-GO. The morphological investigation confirmed the effect of aniline on the stabilization and dispersion of Pt NPs on the r-GO surface. The f-GO was confirmed by the appearance of a new peak at 399.9 eV, which was assigned to the nitrogen band (N 1s) using XPS analysis. The formation of the Pt NPs and their existence in the hybrid were confirmed by XPS and XRD analysis. Four-point probe investigations revealed higher electrical conductivity and lower sheet resistance of the Pt NPs/r-fGO hybrid prepared using the aniline stabilizer compared to the same weight percentage of the Pt NPs/r-GO hybrid without aniline. The Pt NP loading on the surface of r-GO with uniform dispersion has a great effect on the electrical conductivity. The enhancement of the electrical conductivity of the hybrid prepared using the aniline stabilizer originated from the morphological structure, small particle size, uniform dispersion in large quantities of Pt NPs and good interfacial interaction between the Pt NPs and r-GO hybrid. Consequently, the resistance decreased in the case of highly dispersed Pt NPs on the surface of the r-GO using the aniline stabilizer.
